# Partners in Recovery: A Case Study of a National Support Coordination Program for People with Severe and Persistent Mental Illness

**DOI:** 10.5334/ijic.9129

**Published:** 2026-05-20

**Authors:** Jennifer Smith-Merry, Joel Hollier, Nicola Hancock, Bill Gye, Luis Salvador-Carulla, Kieran Halloran, William Campos, Sebastian Rosenberg

**Affiliations:** 1 Centre for Disability Research and Policy, University of Sydney, Australia; 2 Independent mental health advocate, Australia; 3 Health Research Institute, University of Canberra, Australia; 4 NCEPH, Australian National University, Australia; 5 Social Work Supervisor and Mental Health Services Coordinator, Australia; 6 Independent Community Living Australia, Australia; 7 Brain and Mind Centre, University of Sydney, Australia

**Keywords:** Partners in Recovery, psychosocial disability, mental health, care coordination, NDIS

## Abstract

**Objective:**

Partners in Recovery (PIR) was an Australian Commonwealth Government-funded program supporting 35,000 people with complex needs, who experienced severe and persistent mental illness (SPMI). The program was designed to foster integrated care to address fragmented and missing supports. Internationally it is a rare example of a national coordination program evaluated in multiple local contexts, from multiple stakeholder perspectives. This paper examines factors that contributed to the program’s strengths and weaknesses, contextualising this in relation to the limits of subsequent supports.

**Methods:**

This case study draws together 30 program evaluation papers, identified through a range of search strategies. Utilising Arksey and O’Malley’s review framework we collaboratively developed a synthesis of themes and findings.

**Results:**

The support facilitator role was essential to implementation as was organisational environment. As a cornerstone of care for people with SPMI, support coordination required *effective collaboration; strong communication; individualised, flexible, and recovery-oriented support;* and a *well-equipped workforce*.

**Conclusions:**

Data from multiple evaluations of PIR demonstrate the importance of care coordination for SPMI which is underpinned by a recovery-oriented key worker, localised approaches and flexible funding. These are key attributes of integrated support which can inform practice and policy development for this group internationally.

## Introduction: Locating Partners in Recovery Within the Australian Policy Landscape

The Australian mental health system has long been plagued with fragmentation, characterised by a failure to join up those services and supports needed to meet the needs of people with severe and persistent mental illness (SPMI) [1,2]. One key element of this failure has been the split in overall responsibility for mental health, whereby the Australian (federal) government funds primary mental health services while the state and territory governments fund hospital based acute and outpatient care. Community mental health services, especially psychosocial services, have also been poorly funded and neglected in relation to mental health policy development so have not developed at a rate that has kept up with need [3]. In 2013, the Australian Government assumed a new level of responsibility for community mental health care, choosing to fund several new programs, including Partners in Recover (PIR). The overarching aim of the PIR program was to facilitate the integration of care through the coordination of services (both clinical and non-clinical) for 35,000 people with SPMI who were experiencing complex needs which were not being met by existing services [4].

With its person-centred focus, care coordination “continues to be endorsed as a key component within modern mental health systems worldwide” [5]. Care coordination models have proliferated across the international mental health policy landscape, with a variety of models found throughout the United Kingdom, New Zealand, the Netherlands, the United States, and elsewhere [5,6]. Following the process of de-institutionalisation, and in response to the increasing complexity of community-based and in-patient services offered, an urgent need for integrated care emerged. Despite this emphasis, there is little evidence of de-siloing of systems in most jurisdictions. A meta-analysis of global care-coordination research has concluded that “a gap has existed over several decades between policy aspirations for personalised care planning and coordination and the realities of everyday practices and everyday experiences of service users and carers” [7]. While approaches and definitions differ, care coordination is an approach to integrated care which puts the individual at the centre of the system and works with them to coordinate services around their needs. This differs from approaches aimed at integrating the system level [7]. PIR provides a well evaluated example of a successfully implemented national care coordination approach for people with SPMI. It continues to be hailed as a successful model of coordination that is able provide integrated care in the context of complex needs related to SPMI [e.g. 8,9]. Because it is a rare example of successful integrated care evaluated in multiple divergent sites, a scoping review of its operation provides important evidence that may be used to develop flexible, national care coordination-type programs internationally [10].

PIR was designed to operate nationally via consortia of government and non-government organisations in each local Primary Health Network (PHN) (originally Medicare Locals, before these were replaced by PHNs). PIR was originally implemented through the federal Gillard Labor government by then Mental Health and Ageing Minister (current Health Minister) Mark Butler and designed to be rolled out in each Medicare Local region, under successive funding rounds. While intended to operate nationally, due to a change of government, the PIR program was not extended to other regions beyond the first round of funding, which meant there were a small number of regions in which it was not offered. Mandatory for each consortium was the use of ring-fenced funding for program evaluation. Many consortia engaged academic research teams to undertake the evaluation, which has meant that there is a rich amount of data available reflecting on PIR implementation and outcomes.

Central to the PIR program was the role of the “support facilitator” (SF) who worked closely with a client to understand their recovery goals and coordinate services in an integrated manner around these goals. The SF was tasked with being highly collaborative, ensuring an appropriate array of support services were in place for their client, spanning specialist mental health services, legal assistance, general practitioners, housing, employment, education, and community services [11]. This requires a “whole system” perspective which necessitates an understanding of the local ecosystem of services [12]. While clear participant targets and regional funding was determined centrally by the Federal Department of Health and Ageing, each of the PIR consortia developed locally adaptive approaches to rolling out the PIR model. Beyond care coordination (framed as support facilitation under PIR), flexible funding was available to be used for local innovations, including small group programs for PIR clients and the broader community, small research projects and, in a limited way, to purchase bespoke support for individual needs, where it was otherwise not already available in existing systems. SFs worked within individual consortia partners but were part of a team collaborating across consortia. These teams were seen as a key success factor in the program. Other roles within the consortia were team leaders, conceptualised as senior SF roles, and peer workers [13,14].

PIR operated through a no-wrong-door approach – receiving referrals from any source – which would then be assessed based on the target population. An example of numbers of referrals and participants in an individual area comes from the evaluation of PIR in the Western Sydney region, which received 1639 referrals over the period November 2013 to 31 March 2016 [10]. Of these 1363 were accepted as PIR participants. The most common diagnoses of PIR clients in that setting were schizophrenia and delusional disorders (36%) and mood disorders (25%). The most common ‘complexities’ related to physical ill-health (58%), drug and alcohol problems (53%), physical disability (33%) and intellectual disability (18%).

Despite local evaluations evidencing PIR’s success, [15,16] the PIR program started to be wound down with the roll out of the National Disability Insurance Scheme (NDIS), with services subsumed with varying degrees of success into the NDIS. PIR ceased entirely in 2019. Since then, people with SPMI (framed as ‘psychosocial disability’ by the NDIS) have continued to struggle navigating an increasingly complex web of services [17]. As a group, people with SPMI have failed to receive consistent psychosocial support in a landscape now dominated by the NDIS. The NDIS provides lifelong, individual support via personal budgets to eligible participants who can then use those budgets to purchase supports in areas related to their needs which will support their “independence and social and economic participation” [18] (it does not cover health supports). The average annual budget available to each psychosocial disability participant is AU$78,000 [19]. Despite this significant investment and these promising aims, there is a lack of clear evidence that the NDIS is meeting the needs of the majority of people with SPMI [20]. As of December 2023, the Scheme supports 63,508 people with a primary diagnosis of ‘psychosocial disability’, compared to the estimated 686,000 people with SPMI in Australia [20,21]. Many previous PIR participants have not been accepted into the NDIS. NDIS participant outcomes data is very limited. However, people with psychosocial disability under the NDIS do not appear to enjoy the same outcomes as people with other disabilities. For example, the social and community engagement rate of people with psychosocial disability is 30%, compared to 41% for all participants, with an average rate of improvement of only 4% from initial assessment. This compares with a 49% engagement rate and 11% increase from baseline for intellectual disability. The employment rate (any employment) at initial assessment is only 11% for psychosocial disability compared to 22% all participants, with an average rate of improvement of 0%. For intellectual disability this compares to an initial 27% employment rate and 1% increase.

The factors underlying the limited eligibility and lack of outcomes for people with psychosocial disability/SPMI are multiple but include the need for participants to prove disability ‘permanence’ to meet NDIS access criteria, and failure to clearly identify and provide access to evidence-based supports [22,23]. This contrasted with the conditions of access under PIR. Whereas PIR was available to anyone with SPMI for a time-limited period, the majority of people with SPMI cannot meet the NDIS permanence criterion. This means that they are outside the NDIS, but, because of the discontinuation of other psychosocial support programs such as PIR, also unable to access integrated supports through the mental health system.

Four years after the final closure of PIR, and 10 years after NDIS commencement, the major 2023 review of the NDIS concluded that “a new psychosocial disability approach is needed to focus on personal recovery and better connect the NDIS with the wider ecosystem” [24]. To foster this connection, a ‘Navigator’ role has been proposed, tasked with helping participants to “identify evidence-based supports to live the life they want to lead, and to connect with mental health services, education and employment.” As this role is formulated and changes are considered, we can learn from evaluations of previous support coordination models to inform a new approach to addressing psychosocial disability for people with SPMI.

Furthermore, given the global interest in recovery-based programs and the unique nature of Australia’s policies and scope of evaluation in this space, there is burgeoning international consideration of services like PIR. As Isaacs and Farhat contend, “a care coordination model is feasible and appropriate in most developed nations, and its implementation has the potential to overcome the challenges of recovery-oriented services designed for individuals with SPMI” [25]. Given the mandatory evaluations conducted of the Partners in Recovery Program, it is appropriate that a scoping review of the literature synthesise findings and draw together themes that can assist in the development of such care coordination models. With the above context in mind, the research questions this paper seeks to address are:

1. What are the factors that contributed to the Partners in Recovery Program’s strengths and weaknesses?
2. What lessons can be learned from the Partners in Recovery Program that can help inform the design and implementation of future support coordination models for people with severe and persistent mental illness?

Lessons drawn from the literature are offered here with the hope they will help inform a strongly evidence-based, recovery oriented, collaboratively developed path forward.

## Methods

The aim of this evidence synthesis of the Partners in Recovery program was to inform both academic understanding of successful national care coordination and support models, and ongoing psychosocial disability practice and policy development both in Australia and internationally. As a widely evaluated program, a significant number of peer reviewed, scholarly articles had been published and as such, a scoping review methodology was adapted, guided by the methodological framework outlined by Arksey and O’Malley [26]. This includes 1) Identifying the research aim and question; 2) Identifying relevant studies; 3) Study selection; 4) Charting the data; and 5) Collating, summarizing, and reporting the results. For clarity and transparency of reporting, the PRISMA-ScR (Preferred reporting Items for Systematic Reviews and Meta-Analyses extension for Scoping Reviews) checklist was used. [27,28] Scoping reviews are utilised to determine what is, or is not yet known about a topic. While this integrated care case study does not constitute a pure scoping review per se, this methodology was adapted as a means of ensuring rigour and clarity in analysis.

Among the paper authors are people with lived/living experience of mental illness. The project from which this research has arisen is guided by an advisory group which includes people with lived/living experience of mental illness, practitioners, government policy makers and academics. The advisory group provided broad feedback on the review design and outcomes, and how we have interpreted the data. Authors played a variety of roles in the PIR program, ranging from no role through to involvement in program design, implementation and evaluation in individual Partners in Recovery locations.

### Data sources and study selection

**Figure 1:**
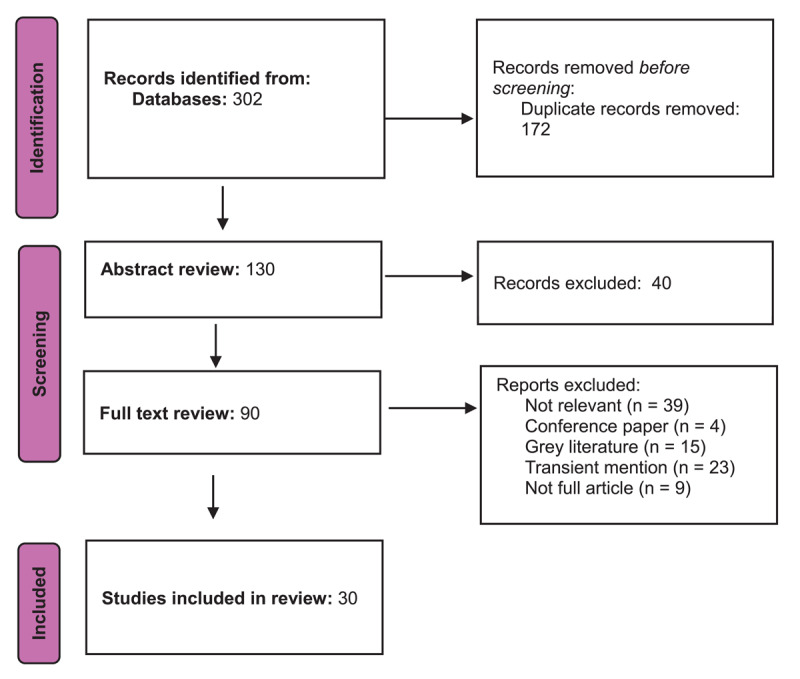
Article sourcing and selection.

Figure 1 outlines the process of article selection as guided by the PRISMA-ScR process. Searches were conducted on a range of databases (Scopus, Web of Science, APA PSychInfo, Ovid, Medline, Embase, and Cinahl) using the terms “Australia* AND “partners in recovery”.

Searches returned 302 articles published between 2011 (when PIR was proposed) and July 2024 (when search was conducted). Duplicates were removed, and articles that did not directly report on PIR outcomes and implementation, or that only discussed PIR in passing were excluded from the study based on discussion between the first two authors.

Final inclusion criteria included:

– Published in English
– Covered the relevant Partners in Recovery program (the phrase has been used to a limited extent in other non-Australian settings)
– Was a full peer-reviewed journal article

After accounting for the above criteria, 30 articles were selected for further analysis to inform the case study.

### Data extraction

Following Arksey and O’Malley’s [26] framework, charting the data involved a process of extracting study participant details, study methods, PIR participant outcomes, implementation barriers and facilitators, and broader system outcomes (including notes about NDIS and the PIR consortia model). These data points were deemed most useful to answer the study’s overarching questions and are summarised in Appendix. This process was conducted by two researchers, JH and JSM.

### Data synthesis and analysis

Analysis of the resulting data was conducted by JH with support from JSM. The data were coded and themes were developed inductively according to the research questions. These themes were then brought together and the main themes identified in discussion between authors.

Arksey and O’Malley also suggest a “‘consultation exercise’ to inform and validate findings from the main scoping review” [26]. To corroborate, clarify, and build upon findings, a draft version of this paper (including a summary of findings) was distributed for comment to a group of researchers and practitioners who were involved in the Partners in Recovery program. These individuals had variously held management, design, implementation, and evaluative roles within the program. Comments received from this group served to provide further nuance and clarity to what was provided within the peer-reviewed literature.

## Results

### Overview of Studies

Thirty papers were identified for inclusion, including twenty-four empirical data studies, three commentaries, one case study, and two literature reviews. Empirical data studies incorporated surveys (6), interviews (17), creative qualitative data (1) webpage and document analysis (4) focus groups/workshops (3) referral data (1) and standardised assessment results (4). Literature reviews were incorporated as a way of placing the program in its broader context.

Appendix provides a summary of studies, including data extracted according to the methodology above.

### Participant Outcomes

Seven studies were identified that directly reported participant outcomes (five quantitative components (n = 1332 participants across all studies), four qualitative semi-structured interview components (n = 85 participants) [4,29,30,31,32,33,34]. Studies consistently reported positive outcomes for participants in the PIR program. For example, Isaacs et al.’s [33] longitudinal study (n = 508 participants) found significant decreases in unmet needs of psychological distress (89% baseline – 27% follow up), daytime activity (72% – 22%), and company (67% – 22%). Hancock et al (n = 703) also identified positive outcomes from baseline to follow up within these domains. In their comparison of needs at intake and follow up Hancock et al. found that recovery scores measured using the RAS-DS (n = 261) improved an average of 5% across each recovery domain, with the biggest increases in relation to the statements: “I can handle what happens in my life”, “I have my own plan for how to stay or become well” and “I have a purpose in life” [4].

Gulliver et al. [29] (n = 25 participants) found that “participants’ perceptions of recovery and their confidence in the health system also improved over both follow-up time points in the study,” while Banfield and Forbes [30] report that 100% of study respondents (n = 25) had a positive response to collaborative goal setting (i.e. they felt central to the recovery oriented care).

Stewart et al. [31] provide a helpful thematic framework that summarises the essential elements leading to the high rate of positive participant outcomes. They report that *empowerment* (capacity to influence the participants’ care team), *transformation* (improved mental health), *and enhanced social connections* (reduced isolation and loneliness) were all vital ingredients in the success of the program. Overall, participants felt supported, emerged with a sense of hope, achieved greater clarity and order in their lives, reconnecting and maintaining social contacts, and overcoming a wide range of challenges [32].

### Costs and Funding

As this case study sought to understand strengths and weaknesses of Partners in Recovery, it was appropriate to consider a cost analysis of the program. Only one study explicitly analysed the costs of a local PIR program. Evaluating the Gippsland PIR, Isaacs et al. found that “The total cost of providing the service for a consumer per year (set-up and ongoing) was estimated to be AUD$15,755 and the ongoing cost per year was estimated to be AUD$13,434…. The potential cost of not adequately supporting such persons is likely to far outweigh that of doing so” [35].

### Implementation Barriers and Enablers

Fourteen studies reported explicitly on a range of barriers and enablers to implementation of the PIR program. This was made up of three quantitative components (n = 1215 participants across all studies), thirteen qualitative components (n = 596 participants), and one systematic webpage content analysis. Across these studies five key barriers to implementation were identified: system siloing, communication barriers, role and procedural confusion and service gaps. Exploring these barriers and enablers was particularly beneficial in answering question two of this review regarding lessons learned that may inform future support coordination programs.

*System siloing:* Given the goal of enhancing integrated care for service users, the extent to which this was not present was readily apparent in the literature. System siloing was particularly evident between clinical and social services and became a barrier to implementation when SFs encountered resistance to entrenched practices [34]. This was exacerbated by a sense of competition across the sector [14]. Siloing was particularly evident when diverse services held different cultures, workflows, and understandings of mental health [36,37].

*Communication barriers:* Given the central nature of communication to the care coordination model, the difficulty of maintaining adequate flow of information was frequently mentioned [14,30]. This ranged from getting the message out about the program [34], communication between service providers [30,31], insufficient IT infrastructure [30,34], an unwillingness to collaborate across jurisdictional boundaries [38], and a lack of understanding passed on about participants’ histories [30].

*Role and procedural confusion:* The unique and novel nature of PIR mean that implementation was hampered by a lack of understanding regarding the nature of the program, and the role and expertise of SFs [14,38]. This was particularly felt when there was a lack of formalised agreements between PIR and clinical teams [30], or when there were unclear policies and procedures for SFs to follow [34]. For peer workers (employed with the criteria of lived experience of mental illness), this uncertainty was exacerbated by the daily stressors of the role, which in turn threatened to challenge their own recovery journey [13].

*Service gaps:* Coordinating services became impossible when there was a lack of services to coordinate [30,39]. Furthermore, rural services were limited, with most services concentrated around hospitals and town centres [1,40]. In a relatively well-serviced area (Western Sydney) there was a lack of alternatives to hospitalisation, along with day care services and employment options [1]. Finaly, a key service gap identified was housing, which had further ramifications. When people lacked adequate housing they were less likely to engage fully with PIR [31,41], and data suggests they had difficulty getting on to the program [34,41].

Facilitators to implementation included the right staffing, effective collaboration, clear communication channels, individualised, flexible, recovery-oriented support and stability and security of foundational supports external to PIR.

*The Right Staffing:* The SF role was viewed as a complex position requiring a unique skillset and knowledge base. In order for the PIR program to be a success, participants had to feel as though the SF was a good “fit” [31,42]. This meant that the SF had to genuinely care about the client, with a supportive, respectful and responsive relationship [31,36,42]. In particular, SFs with diverse backgrounds were highly regarded [14]. Smith-Merry et. al [14] described the diversity of SFs backgrounds including “nursing, psychology, homelessness services, disability, psychology, policy, refugee advocacy and social work” and that “these backgrounds give them a broad range of skills” which was more important than a qualification itself. In one study, SFs were required to have a tertiary qualification [13], however this was not the case across different Medicare Locals.

Exploring this role further, it was noted that key competencies for the SF included: reliability, a strong ability to collaboratively develop action plans, good listening skills, strong connections, relatable, recovery oriented, dedicated, non-medicalised, holistic, flexible, resourceful, a good understanding of local services and resources, strong administrative skills, organised, discerning, creative, high level social skills, strong communication, respectful, and genuine [14,30,42,43]. This long list of competencies reflects the complexity of the role. Sutton et al. provided a summary of the seven key areas the SF entailed, including:

“1) Being a single point of contact for clients and other services; 2) care coordination; 3) assisting the client to become self-reliant; 4) achieving good outcomes for clients with confronting behaviours; 5) judiciously using flexible funding; 6) clearly outlining their role with clients and maintaining boundaries; 7) and performing a different role from that of the mental health case manager” [43].

Alongside the SF role, two studies mentioned the impact of Peer Workers employed within local consortia to work alongside SFs. Horsfall et al. found that peer workers facilitated better engagement among clients and services, and made services more accessible [37]. There was, however, recognition that lived experience alone was not sufficient and some peer workers reported needing other training and qualifications [13].

*Effective collaboration:* Collaboration within PIR entailed a complex network of relationships. In order for successful implementation of the program, SFs needed the ability to collaborate with their clients, with other SFs, with service providers, and with other stakeholders [36,41,42,44,45]. Collaboration between SFs entailed opportunities for SFs to exchange background knowledge and was a key driver for positive outcomes, and for the establishment of local knowledge [38]. The role of “boundary spanner” as a distinct position tasked with creating systems change was highly valued, and sector bridging initiatives were seen as vital [39,41]. Finally, co-creation alongside people with lived experience was described as a non-negotiable which required a significant time commitment, but ultimately saved time thanks to a collaborative approach [44].

*Clear communication channels:* As an element of effective collaboration, clear communication channels were highlighted as key to service implementation [36,41]. This also took the form of good governance, incorporating clearly articulated roles, formally established partnerships, and role clarity between organisations [46]. Clear communication also entailed active recruitment of potential clients who may be located in hard-to-reach communities to ensure intended populations were appropriately informed regarding PIR [47].

*Individualised, Flexible, Recovery-Oriented Support:* Individualised care was seen as critical, ensuring that SFs tailored services and interactions to consumer’s needs [42]. In embedding an individual focus to the PIR, a recovery-oriented framework was essential [34]. As Trankle et al. noted, this recovery focus was operationalised right across the program from national PIR policy documents through to partnerships with community representatives [34]. Flexibility was also seen in how flexible funding was used, ensuring SFs and clients could collaboratively make best use of funds to broker needed supports where not freely available [30].

*Stability and Security of Foundational Supports:* As established above, without key services, the PIR could not function effectively. This was particularly seen in regard to housing which served as a basis for stability and security for people experiencing SPMI [41]. Non-clinical provisions (such as housing) were therefore seen as the “foundation” on which recovery could then be prioritised [46]. As such, when there was stability and predictability of these foundational services, clients could expect better outcomes of the program.

### Developing an effective support coordination model for integrated care

This case study also provides insight into the elements that contribute to a positive support coordination model for the sake of integrated care. Within the PIR literature, support coordination was reflected on from both a structural, and a personal level, and integrated care was perceived of as crucial. As the SF played a central role in PIR, there was significant attention paid to how the role was adopted/adapted, essential criteria for the role, and what made the role successful, as described above. Key structural ingredients for the development of an effective support coordination model were the PIR consortia and networks: PIR was a nationally defined program, implemented by a range of consortiums (largely Medicare Locals) across Australia. Within each of these consortiums, individual organisations (often NGOs) provided the framework in which they operated. These organisations and consortia were therefore key to the SF role because they provided the organisational structure sitting around this key role. This meant that SFs had to comply with policies and directions from multiple sources (consortium and local organisations) which could be confusing [14]. Practically, in order to develop the SF directives, consortium members came together to discuss, shape, and negotiate roles, as well as foster collaboration [34,36]. This was a complicated process and necessitated clear communication drawing on prior sector-based knowledge within the consortium and individual organisations [36].

In order to further develop networks SFs engaged in interagency forums [36]. These interagency forums were often based around a particular topic (eg. housing needs), with guest speakers or collaborative knowledge sharing [14,36,38,48], and where possible members of SF teams attended different interagency groups to report back findings [14].

*Continuity of care:* SFs spent significant energy attempting to achieve continuity of care. This was evident with transitions within and across programs when a lack of collaboration was present, or with the introduction of a new SF or service [30,31]. Stability and security across the realms of material, interpersonal, and environmental needs was seen as a core indicator for positive engagement in the PIR program [46].

### Toward the NDIS, Systems Changes, and Closure of PIR

As the national roll out of the NDIS commenced and the future of PIR became unclear, six studies sought to make recommendations to ensure learnings were integrated into the emerging Scheme [14,17,31,35,47,49]. While SFs were important at drawing the system together around individual needs, PIR did not fix the broader systems integration problems that made this necessary, with longer term change required from social care and health organisations to emphasise collaborative practice [29]. As such “the program’s lasting effects on the system also seemed unclear” [30]. At the conclusion of the Canberra PIR, Gulliver et al. found there was an increased connectivity between services and PIR, however there was not a significant increase between services more broadly [29]. The organisational distress in the NGO sector and problems of the transition of Psychosocial services from PIR to NDIS was identified in the ACT when NDIS was implemented in 2016 and persisted four years later [50].

Reflecting on PIR in the context of the NDIS, it was argued that a dedicated role which worked collaboratively with clients and stakeholders was vital [49]. Highlighting this, Smith-Merry et al. recognised “increasing spaces to be filled by accented SF type roles in, for example, managing money and buying in, finding or negotiating access to services” [14]. In 2019, with the roll out of the NDIS substantially underway, Rosenberg et al. noted that “among the assumptions underpinning the NDIS was that many PIR and PHaMs clients would be transferred to the NDIS. This does not appear to be happening, leaving people without services” [17]. It was also anticipated that some of the accessibility barriers experienced for some groups accessing PIR would continue under the NDIS, and it was recommended that assessment processes consider the diverse needs of groups experiencing vulnerabilities [31].

## Discussion

This scoping review has provided insight into the factors that contributed to the successes and weaknesses of the Partners in Recovery Program, while synthesising themes that could contribute to ongoing policy development. As we struggle with how to address the difficulties of a psychosocial support landscape dominated by a NDIS that both leaves some people with SPMI without support and provides support that lacks effectiveness to others, it is important to consider the learnings from national strategies that have aimed to support this group in the past. This discussion is relevant beyond an Australian context because of the ongoing difficulties faced by many countries internationally that attempt to develop national systems of coordinated care for people with SPMI.

This synthesis of literature has identified that PIR could be considered an effective coordinated care model for supporting the psychosocial support needs of people with SPMI. The key aspects of this model which enabled effectiveness were 1) the relationship between the SF and participant, 2) the focus of this relationship being on the coordination of services and supports to meet individual needs, 3) the organisational environment and consortium structure sitting around the SF, and 4) flexible adaptation to local context.

The relationship between the SF and participant was the key element in supporting recovery for PIR participants. Elements of the relationship most important were a foundation of respect, a genuine sense of care, and a high level of capability to respond within the recovery model with a sound knowledge of appropriate local services. The SF needed an understanding of the operationalisation of recovery, with experience more highly valued in the SF role than qualifications. The organisational environment in which the SF was situated was also important as a foundation for this relationship. Environment referred to both the worker’s immediate supervising organisation and the consortium structure implementing PIR in individual regions. These provided connections and support for the SF.

With the disappearance of PIR no SF-type roles currently exist for those not eligible for the NDIS in Australia. The NDIS will fund support coordinators or ‘recovery coaches’ but these two roles differ from what was previously offered under PIR in several ways which may be why they do not result in the reduction in needs experienced by PIR participants. Neither role must be embedded within an organisational environment focused on recovery and broader mental health support, which means that they are not supported by either the broader organisational capacity of a host organisation (and the broader consortium network), or the team of SFs working around them. While workers taking on the recovery coach role must have a qualification or experience working in mental health, support coordinators do not need any experience in mental health. This means that coordination can occur without being based within the principles of recovery, and resulting coordination made without a good understanding of the existing mental health supports needed. Previous evaluations of care planning in other contexts have shown that knowledge of mental health and the resources and supports available in the sector are essential for effective care planning [51]. To improve the operation of the NDIS it is therefore important that these two criteria are met for all coordinators: 1) existing understanding of the sector and mental health and 2) a supportive organisational structure which also has good understanding of these things and has established cross-organisational collaborations. This is also important for current reform of support for people who are not eligible for the NDIS, engaging with the ‘foundational supports’ currently being developed and implemented in states and territories.

Key barriers to the implementation of PIR were communication, siloing of services, and role confusion. These are systemic factors that lead to poorly integrated and coordinated care and indeed were part of the systemic failures that made a PIR approach necessary in the first place [14]. The positive outcomes for clients within the relatively short lifetime of the PIR program proves these entrenched systemic problems can be sufficiently overcome through the other key aspects of the program, primarily the key worker and organisational structures sitting around them.

## Conclusion

Lessons learned from the PIR program call for an individualised, collaborative care coordination model built on, and by, a responsive, relational, respectful workforce.

Australia is now grappling with impending changes to the NDIS, in response to concerns about systemic problems and a lack of support for people with SPMI both within and outside of the NDIS. Concurrently, ongoing global interest in recovery-oriented models of care present profound opportunities to shape the next chapter of psychosocial disability policy. Learnings gleaned from this case study offer a potentially low-cost approach which has shown proven benefits for people with SPMI. This is important evidence for the design of foundational supports and the new NDIS Psychosocial Early in Scheme Program for people entering via s25 of the NDIS Act [24].

The richness of the evaluation data that the review was able to draw on also underscores the importance of implementing national evaluation strategies that allow us to reflect on past policy impacts to enhance evidence informed decision making and improvements to complex programs to support for people with severe and persistent mental illness.

## Additional Files

The additional file for this article can be found as follows:

10.5334/ijic.9129.s1 Appendix. PIR Abridged Summary of Studies.
